# Not All Wnt Activation Is Equal: Ligand-Dependent versus Ligand-Independent Wnt Activation in Colorectal Cancer

**DOI:** 10.3390/cancers12113355

**Published:** 2020-11-13

**Authors:** Sam O. Kleeman, Simon J. Leedham

**Affiliations:** 1 Cold Spring Harbor Laboratory, Cold Spring Harbor, NY 11724, USA; skleeman@cshl.edu; 2 Intestinal Stem Cell Biology Lab, Wellcome Trust Centre Human Genetics, University of Oxford, Oxford OX3 7BN, UK

**Keywords:** Wnt, signaling, colorectal, cancer, porcupine, R-spondin, serrated, immunotherapy

## Abstract

**Simple Summary:**

Colorectal cancer is the third most common cause of cancer-related deaths. The Wnt signaling pathway is activated by genetic mutations in most patients with colorectal cancer. A number of different types of Wnt pathway mutation have been described: some increase the sensitivity of tumor cells to Wnt ligands produced by stromal cells (ligand-dependent), while others drive downstream activation of the pathway (ligand-independent). Ligand-dependent tumors are of particular interest as there are a number of emerging treatment options, such as porcupine inhibitors, that can specifically target these tumors. In this review, we discuss what is known about these different types of Wnt activating mutations. We propose that ligand-dependent tumors should be viewed as a separate subset of colorectal cancer with its own biomarkers, prognosis and targeted therapies.

**Abstract:**

Wnt signaling is ubiquitously activated in colorectal tumors and driver mutations are identified in genes such as APC, CTNNB1, RNF43 and R-spondin (RSPO2/3). Adenomatous polyposis coli (APC) and CTNNB1 mutations lead to downstream constitutive activation (ligand-independent), while RNF43 and RSPO mutations require exogenous Wnt ligand to activate signaling (ligand-dependent). Here, we present evidence that these mutations are not equivalent and that ligand-dependent and ligand-independent tumors differ in terms of underlying Wnt biology, molecular pathogenesis, morphology and prognosis. These non-overlapping characteristics can be harnessed to develop biomarkers and targeted treatments for ligand-dependent tumors, including porcupine inhibitors, anti-RSPO3 antibodies and asparaginase. There is emerging evidence that these therapies may synergize with immunotherapy in ligand-dependent tumors. In summary, we propose that ligand-dependent tumors are an underappreciated separate disease entity in colorectal cancer.

## 1. Introduction

Metastatic colorectal cancer (CRC) is a lethal malignancy with a five-year survival of less than 15% [1]. Patients with metastatic CRC are treated with combination cytotoxic chemotherapy alongside monoclonal antibodies targeting angiogenesis or epidermal growth factor receptor (EGFR) [2]. There is a need to develop new therapeutic strategies for metastatic cancer, especially in light of evidence showing rapid increases in CRC incidence affecting younger patients [3]. Molecular profiling of CRC has shown considerable disease heterogeneity, suggesting that patients might benefit from precision medicine, in which treatments are personalized for their tumor profile [4]. For example, immunotherapy targeting PD-1/PD-L1 signaling is only active in hypermutated tumors, while anti-EGFR antibodies are only effective in tumors without downstream mutations [5,6,7].

Colorectal cancer is characterized by near-ubiquitous activation of the Wnt signaling pathway [8]. The Wnt pathway is an evolutionarily conserved mechanism for intercellular communication, with essential roles in embryogenesis and adult tissue development [9]. In the colonic crypt, Wnt signaling is necessary to maintain the adult intestinal stem cell niche and epithelial homeostasis [10]. Colorectal tumors are dependent upon aberrant Wnt signaling to maintain stemness and a de-differentiated phenotype and genetic Wnt inhibition leads to rapid tumor regression [11]. Additionally, Wnt signaling can protect cells from immune surveillance, thus restricting anti-tumoral immunity [12,13]. Altogether, this suggests that the Wnt pathway could be a viable therapeutic target for patients with CRC.

Colorectal tumors are thought to evolve through the sequential acquisition of mutations driving progression from a normal founder cell to adenoma and then carcinoma [14]. Adenomas can be histologically classified as either conventional, such as tubular or tubulovillous (TVA), or serrated, such as sessile serrated lesions (SSL) or traditional serrated adenomas (TSA) [15]. Serrated adenomas are characterized histologically by a saw-tooth morphology. The cell-of-origin for TVA is likely the crypt-based columnar stem cell [16] while the cell-of-origin for serrated lesions is unknown, but may derive from ectopic crypt foci in the rare traditional serrated adenoma subtype [17].

Here, we present evidence for a new model of CRC in which Wnt pathway activation can take one of two distinct trajectories, ligand-dependent (LD) and ligand-independent (LI), with implications spanning tumor biology, screening, diagnosis and treatment. We will first outline the Wnt signaling pathway in the normal colon and types of recurrent Wnt mutations in CRC. We will then discuss morphological and molecular biomarkers that can be used to identify LD tumors in the clinic. Finally, we will argue that this model has the potential to transform the landscape of precision medicine in CRC.

## 2. Wnt Signaling Pathway

**Figure 1:**
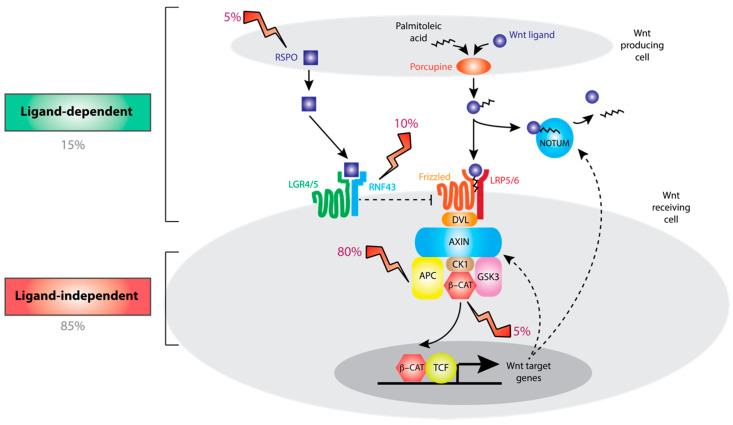
Overview of Wnt signaling pathway. Wnt ligands secreted from stromal cells are activated by porcupine-mediated post-translational modification and bind to Frizzled (FZD) receptors on Wnt receiving cells. This functions to inhibit a destruction complex containing axin-like protein (AXIN)1/2 and adenomatous polyposis coli (APC) thus disinhibiting β-catenin (CTNNB1), the master transcriptional regulator of Wnt singaling. Frizzled receptors are degraded due to the action of ring finger protein 43 (RNF43), which is in turn inhibited by binding of R-spondin (RSPO) ligands to leucine-rich repeat-containing G protein-coupled (LGR) family receptors, thus augmenting Wnt signaling tone. Wnt pathway activation is regulated at multiple levels by negative feedback loops, including those mediated by AXIN2 and notum palmitoleoyl-protein carboxylesterase (NOTUM). Recurrent mutations in CTNNB1 and APC result in ligand-independent pathway activation while mutations in RSPO and RNF43 depend upon binding of Wnt ligands to Frizzled receptors. GSK: glycogen synthase kinase; LRP: lipoprotein receptor-related protein.

The Wnt signaling pathway in normal colon crypts is summarized in Figure 1. Briefly, canonical Wnt ligands are secreted by the cells in the stem cell niche following O-acylation by porcupine [18,19]. Wnt ligands bind to Frizzled (FZD) and lipoprotein receptor-related protein (LRP) receptor complexes on the plasma membrane of neighboring cells [20]. Both Wnt ligand secretion and binding to FZD depend upon acylation of Wnt ligands [21]. The downstream effector of Wnt signaling is the transcriptional co-activator β-catenin (CTNNB1). In the absence of Wnt ligands, CTNNB1 is degraded by the action of a destruction complex containing adenomatous polyposis coli (APC), axin-like protein (AXIN1/2), glycogen synthase kinase (GSK3) and casein kinase (CSNK1A) [22]. Wnt ligand binding inhibits the destruction complex, thus stabilizing CTNNB1 and activating expression of Wnt target genes. An additional level of regulation comes from E3 ubiquitin ligases ring finger protein 43 (RNF43) and zinc and ring finger 3 (ZNRF3), which constitutively degrade FZD to repress Wnt signaling [23]. R-spondin (RSPO) ligands bind to leucine-rich repeat-containing G protein-coupled (LGR) receptors, inhibiting RNF43/ZNRF3 and substantially amplifying Wnt signaling [24]. There are four homologous human RSPO ligands (RSPO1-4) and, while all four can bind to LGR-family receptors, the EC_50_ for activation of Wnt signaling varies 100-fold, with RSPO2 and RSPO3 demonstrating the highest potency (0.02–0.05 nM) [25]. R-spondins are produced by stromal cells adjacent to the stem cell niche [26]. Consistent with this, R-spondin signaling is necessary to maintain the stem cell niche, both in vivo and as part of organoid culture systems [24,27,28]. Wnt target genes, such as notum palmitoleoyl-protein carboxylesterase (NOTUM) and AXIN2 are negative regulators of Wnt signaling, functioning as negative feedback loops to fine-tune and limit downstream signaling [29]. AXIN2 is an inducible component of the destruction complex, while NOTUM works in the extracellular space to deacetylate and inactivate Wnt ligands [30].

## 3. Ligand-Dependent and Ligand-Independent Alterations in Colorectal Cancer

Large-scale sequencing studies in CRC have established the presence of pervasive Wnt pathway mutations. Recurrent mutations include loss-of-function mutations in APC and RNF43, and gain-of-function mutations in CTNNB1 and RSPO2/3 (Figure 1, Table 1) [8,31,32]. Consistent with driver mutation status, in vivo modeling indicates that these mutations can be sufficient for colorectal tumorigenesis [33,34,35,36]. For tumor suppressors APC and RNF43, we only consider protein-truncating mutations or deletions as potential driver alterations [37]. ZNRF3 is a homolog of RNF43 but truncating mutations are rare in colorectal tumors, potentially reflecting its comparably low mRNA expression in normal colon and colorectal tumors [8,38]. This is in contrast to the situation in murine intestine, in which Znrf3 and Rnf43 gene expression is comparable, and loss-of-function alterations in both Znrf3 and Rnf43 are necessary to activate Wnt signaling [36,39].

While *APC* and *CTNNB1* alterations drive downstream, constitutive activation of the Wnt pathway that is independent of Wnt ligand binding (ligand-independent, LI), *RSPO* and *RNF43* alterations disrupt the synergistic RSPO axis and by doing so, amplify endogenous and otherwise intact, Wnt ligand signaling (ligand-dependent, LD). APC mutations are characteristically nonsense or frameshift alterations affecting the “mutation cluster region”, often with a second “hit” from loss of heterozygosity [40]. CTNNB1 mutations are gain-of-function missense mutations affecting specific amino acid residues that are phosphorylation sites for components of the destruction complex [41].

RSPO mutations induce R-spondin ligand overexpression either from epithelial cells (autocrine), as RSPO fusion genes [42]. R-spondin gain-of-function is only observed for RSPO2 and RSPO3, consistent with their enhanced potency to induce Wnt signaling in vitro [25]. RSPO3 fusion genes commonly result in the replacement of RSPO3 exon one and promoter with that of a gene with higher basal expression, resulting in a functional epithelial-expressed protein [32]. A wide range of fusion partners have been identified including PTPRK, EIF3E, NRIP1 and PIEZO1 [43,44], all of which are associated with relatively high constitutive gene expression [38]. RSPO fusions cannot be reliably identified even from whole-genome sequencing due to large inconsistency in genomic alterations, while the transcript breakpoints are more stereotypical [32]. Alternatively, in a rare subset of colorectal tumors, we identified R-spondin overexpression in the absence of RSPO fusions or any other detectable Wnt driver alteration [42]. In situ hybridization demonstrated high stromal RSPO3 expression in these tumors, implicating a role for paracrine R-spondin signaling driven by stromal overexpression [42]. RSPO3 overexpression in the absence of gene fusions has been previously detected in lung cancer, where it was associated with RSPO3 hypomethylation [45]. The concept that RSPO overexpression can derive from either epithelial or stromal sources is consistent with previous evidence that RSPO3 expression is significantly and positively correlated with stromal expression signatures [46]. RNF43 mutations are mostly recurrent frameshift mutations at amino acid positions 117 and 659 that result in a truncated gene product [31]. These recurrent mutations occur at tandem repeats called microsatellites whose stability is dependent upon proficient mismatch repair (MMR) [47]. As a result, these mutations tend to occur in tumors with MMR deficiency, detected as microsatellite instability (MSI), which is often caused by promoter hypermethylation of MLH1 [8,48].

Recently, there has been some controversy about whether the RNF43 G659Vfs*41 mutation demonstrably leads to impaired protein function. In vitro transfection experiments have indicated that this mutant RNF43 protein retains the ability to bind R-spondin and repress Frizzled [49]. However, this alteration is associated with significantly reduced RNF43 expression, potentially consistent with nonsense-mediated decay [49], and CRISPR-Cas9 editing of the endogenous RNF43 locus to mimic the G659Vfs*41 mutation, was sufficient to increase cell surface Frizzled expression [50]. Furthermore, the G659Vfs*41 mutation occurs substantially more often than would be expected by chance in microsatellite-unstable tumors, indicating strong positive selection [31,51].

It is important to note that driver Wnt alterations affecting APC, CTNNB1, RNF43 and RSPO in pre-cancerous polyps and tumors show marked mutual exclusivity [42]. There are two logical implications from this: firstly, these alterations are redundantly able to activate Wnt signaling. Secondly, there may be selection against the accumulation of driver alterations in more than one gene. This is consistent with the “just right” theory of Wnt signaling: that there is an optimal level of Wnt activation to drive tumorigenesis. It has been observed that there is a non-random distribution of second “hit” mutations in APC that is consistent with selection for APC genotypes that retain some CTNNB1 repression [52]. Additionally, ectopic expression of R-spondin in APC-mutant mice results in reduced proliferation and increased apoptosis [53], consistent with evidence that Wnt can directly promote apoptosis [54].

## 4. Mutation Selection in Lesion Subtypes

**Figure 2:**
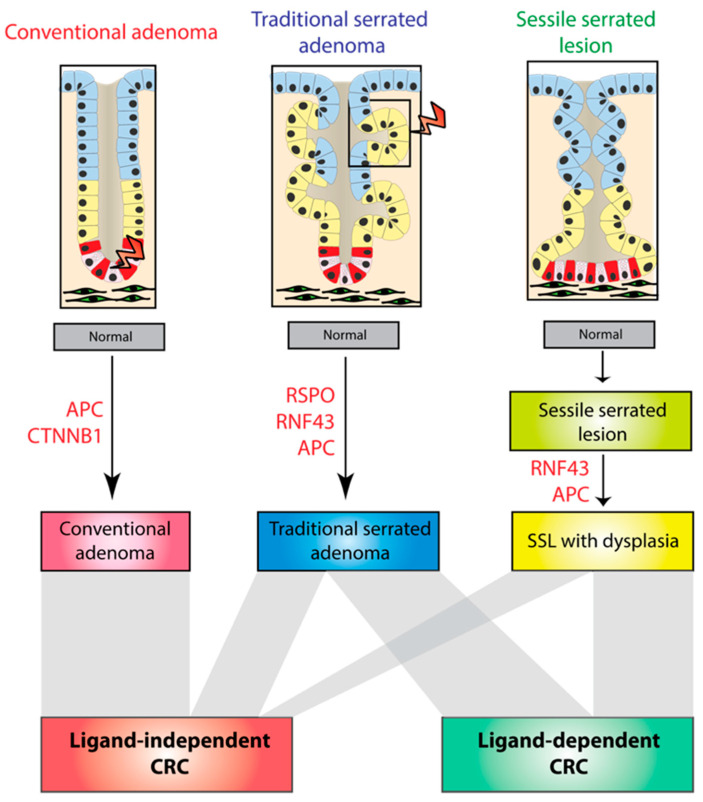
Molecular pathogenesis of different colorectal precursor subtypes. Colorectal cancer develops from three types of pre-cancerous polyps: conventional and serrated adenomas—divided into traditional serrated adenomas (TSAs) and sessile serrated lesions (SSLs). Conventional adenomas are driven by ligand-independent mutations that likely arise in the crypt base columnar (CBC) stem cells. TSAs arise from APC, RSPO or RNF43 mutations, possibly in ectopic crypts. SSL pathogenesis is characterized by the late acquisition of APC or RNF43 mutations, concurrent with the onset of the detectable dysplasia. Ligand-dependent CRC arises from TSAs and SSLs while ligand-independent CRC arises from all three types of polyp (bottom panel).

Molecular profiling in pre-cancerous polyps has shown that ligand-dependent alterations are predominantly seen in the serrated pathway (Figure 2) [44,55]. A total of 55% of TSAs have ligand-dependent alterations, namely truncating RNF43 mutations or RSPO fusions (mostly PTPRK-RSPO3) [44]. In sessile serrated lesions (SSL), mutations in the Wnt signaling pathway are not thought to be initiating lesions as Wnt disruption is observed predominantly in dysplastic rather than non-dysplastic lesions. A total of 50% of SSLs had ligand-dependent RNF43 mutations [55], whereas APC mutations are much rarer in serrated lesions, being detected in 13% and 9% of TSAs and dysplastic SSLs, respectively [44,55]. In contrast, conventional adenomas have a high frequency (>85%) of ligand-independent alterations [56]. APC mutation is sufficient to initiate adenoma pathogenesis [57] and no ligand-dependent alterations have been reported in conventional adenomas [44].

Altogether, this raises the possibility that different intestinal lesions follow distinct molecular carcinogenesis pathways. These different evolutionary trajectories appear to result in the selection of either ligand-dependent or independent mutations. This may also partly explain the mutual exclusivity of Wnt driver mutations discussed above. Why polyp subtypes acquire apparent obligatory Wnt disruption through these different mechanisms is unknown, but may be influenced by the variable cell-of-origin in different lesion subtypes (Figure 2). Indeed, APC mutations induce tumorigenesis in vivo if introduced into the LGR5+ intestinal stem cell but not transit-amplifying cells [16], while RSPO fusions significantly co-occur with loss-of-function mutations in the Bone morphogenic protein (BMP) signaling pathway that are known to induce ectopic crypt formation [58,59]. These data would also suggest that RSPO-mutant colorectal tumors are wholly derived from TSAs.

## 5. Negative Regulation of Wnt Signaling

In some ways, it is surprising that despite multiple levels of negative feedback, ligand-dependent mutations, which act upstream in an otherwise normal pathway, can induce activation of Wnt signaling at all. Ligand-dependent pathway activation would be expected to induce physiological expression of Wnt negative regulators such as AXIN2 or NOTUM, which would function to proportionately constrain activation of the pathway. In contrast, ligand-independent alterations result in downstream, constitutive activation that is uncoupled from the action of negative regulators. We have recently shown that tumors with ligand-dependent alterations are associated with significant repression of Wnt negative regulators, especially AXIN2 [42]. This repression may be at least partly explained by AXIN2 methylation [60]. This raises the possibility that ligand-dependent Wnt activation requires two ”hits”—firstly a driver mutation affecting RNF43 or RSPO, and secondly, epigenetic downregulation of Wnt negative regulators. Indeed, serrated adenomas which are enriched for ligand dependent mutations, have lower AXIN2 expression and increased AXIN2 methylation compared to conventional tubulovillous adenomas [61,62], as do MSI-high cancers that progress via this pathway [45,53]. AXIN2 expression is also decreased in an in vivo model of ligand-dependent tumors, generated by orthotopic engraftment of CRISPR-edited organoids [63]. Furthermore, ectopic expression of AXIN2, leading to re-activation of Wnt negative feedback in an RNF43-mutant cell line (HCT116) resulted in rapid cell death [60,61]. In fact, AXIN2 is not the only Wnt negative regulator known to be silenced by promoter hypermethylation in colorectal cancer: hypermethylation has been detected in negative regulators including WIF1, SFRP1/2/4, DKK1–3 and NOTUM [42,62,64]. These genes are predominantly hypermethylated in ligand-dependent or microsatellite-unstable tumors. This suggests that repression of negative regulators is a more global phenomenon in ligand-dependent CRC, with loss of negative feedback mechanisms at multiple levels of the Wnt signaling pathway.

## 6. Application of AXIN2 as a Biomarker for Ligand-Dependent Wnt Biology

Our finding that ligand-dependent tumors exhibit suppressed expression of negative regulators of Wnt can be harnessed to utilize AXIN2 as a single-gene biomarker to distinguish between ligand-dependent and ligand-independent tumors at the point of diagnosis. This is particularly important as otherwise ligand-dependent tumors would need to be identified from expensive and time-consuming analysis of paired DNA (for APC, CTNNB1 and RNF43) and RNA sequencing (RSPO fusions). Paired DNA and RNA sequencing is simply not practical for routine diagnostic assessment in the clinic, both in terms of cost and the relatively high failure rate of sequencing (>10%) from diagnostic clinical samples [65].

We recently demonstrated that AXIN2 mRNA expression could be used as a discriminatory biomarker with an area under the curve (AUC) greater than 0.93 in three independent cohorts, indicating excellent diagnostic performance. This analysis incorporated both RNA sequencing and microarray profiling to assay gene expression in resection and biopsy specimens [42]. The diagnostic performance corresponded to sensitivity and specificity >90%. We also demonstrated similar results with high-throughput AXIN2 profiling by quantitative real-time polymerase chain reaction (qRT-PCR). These findings were recently supported by the use of an organoid biobank derived from patients with colorectal cancer, in which organoids with RSPO fusions or RNF43 mutations exhibited lower AXIN2 expression than APC-mutant organoids [58]. Our analysis of paired qRT-PCR and immunohistochemistry for AXIN2 showed that there was only weak correlation between AXIN2 mRNA and scored AXIN2 protein expression, suggesting that AXIN2 may undergo significant translational regulation, as has been described previously [66]. This would suggest that profiling of AXIN2 mRNA expression would be the preferred approach to translate this biomarker into the clinic.

It is worth noting that AXIN2 gene expression is widely used as a read-out of global Wnt pathway activation [67] and our findings suggest that this should be interpreted with caution, as AXIN2 expression can be confounded by the type of acquired Wnt disrupting pathway mutation. This confounding has important implications for the interpretation of analyses that have demonstrated inverse correlations between AXIN2 (used as a read-out of Wnt activation) and immune infiltration [13]. Tumors with low AXIN2 expression are enriched with RNF43-mutant MSI-high tumors that have enhanced anti-tumoral immune responses, thought to result from an increased neoantigen load [68]. As a result, the inverse relationship between AXIN2 and immune infiltration may be partly explained by increased mutational load in RNF43-mutant ligand-dependent tumors, rather than reduced Wnt activation.

In summary, the distinction between ligand-dependent and ligand-independent tumors is clinically-actionable because tumors can be robustly discriminated using a low-cost single-gene molecular biomarker.

## 7. Non-Overlapping Clinicopathological Features of Ligand-Dependent Tumors

Consistent with altered Wnt pathway biology and an altered trajectory through the serrated pathway, ligand-dependent tumors have non-overlapping morphological and clinical characteristics with ligand-independent tumors, reflecting an underappreciated separate disease entity in colorectal cancer. Using manual and automated digital pathological approaches, we have demonstrated that ligand-dependent tumors are enriched with mucin [13,42]. Mucin is a high molecular-weight glycoprotein that is secreted by goblet cells and forms a key component of the mucous layer that provides physical protection in the gastrointestinal tract [69]. Mucinous differentiation has long been recognized in a subset of colorectal tumors (around 10%) and is diagnosed in tumors where mucin comprises >50% of the tumor volume [70]. Indeed, mucinous differentiation is associated with microsatellite instability, implicating a link with RNF43-mutant tumors. We have demonstrated that computational-scored mucin area alone could discriminate between ligand-dependent and ligand-independent tumors with an AUC > 0.75. Based on our findings, we propose that mucinous differentiation may well either be induced by ligand-dependent Wnt signaling or reflect the association with the serrated pathway. Consistent with the former hypothesis, the induction of ligand-dependent alterations in organoids is sufficient to generate orthotopic colon tumors with mucinous differentiation [63]. Furthermore, RNF43 mutations in biliary malignancies are associated with mucin hypersecretion [71]. Altogether, this suggests that mucin content, which is routinely scored by histopathologists, can be used as a phenotypic biomarker for ligand-dependent tumors with good diagnostic performance.

In our comparison of ligand-dependent and ligand-independent tumors in a pooled cohort of over 600 tumors with available outcome data, we did not identify any significant differences in prognosis [42]. However, this is likely to mask, considerably, the prognostic heterogeneity between the subsets of ligand-dependent tumors. One way to examine this is to compare specific subsets with their consensus molecular subtype (CMS) classifications, as this study was well-powered to identify prognostic associations incorporating over 2000 patients [72]. Ligand-dependent tumors appear to lie on a continuum between RNF43-mutant tumors which mostly classify as CMS1 (associated with good prognosis) and tumors with stromal RSPO overexpression which mostly classify as CMS4 (associated with poor prognosis). Consistent with this, we observed a high frequency of tumor budding and enriched desmoplastic stroma in tumors with stromal RSPO overexpression, both of which are associated with poor prognosis [73,74]. Of note, mucinous differentiation is associated with marginally reduced overall survival [75]. These data suggest that the prognostic implications of ligand-dependent Wnt biology are likely to be highly heterogenous.

## 8. Selective Vulnerabilities in Ligand-Dependent Tumors

**Figure 3:**
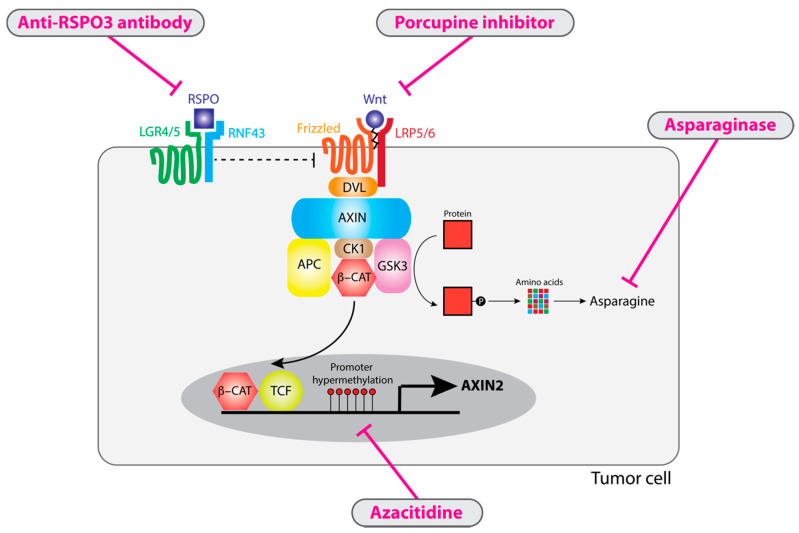
Target therapies for ligand-dependent tumors. All ligand-dependent tumors require Wnt ligands for pathway activation and so are sensitive to porcupine inhibitors that impair Wnt ligand activation. RSPO3 overexpression can be antagonized by anti-RSPO3 antibodies. Ligand-dependent GSK3 inhibition results in reduced proteasomal degradation to generate amino acids such as asparagine, making tumor cells sensitive to asparagine depletion with asparaginase treatment. Ligand-dependent tumors are characterized by AXIN2 repression which can be antagonized with licensed demethylating agents such as azacitidine.

Downstream ligand-independent Wnt signaling has proved difficult to target in solid tumors, reflecting challenges in designing small-molecule inhibitors to inhibit constitutive pathway activation through transcription factors such as beta-catenin [76]. In contrast, from a conceptual and experimental standpoint, ligand-dependent Wnt activation is inherently “druggable” through deprivation of extracellular ligand (Wnt or R-spondin) or attenuation of negative regulator suppression with demethylating agents (Figure 3) [27,60,77]. Furthermore, emerging evidence would indicate that these selective vulnerabilities in ligand-dependent tumors could synergize with immunotherapy targeting PD-1/PD-L1 signaling in tumors [78]. This makes ligand-dependent tumors a fascinating subset of colorectal cancer, with the real possibility of new transformative treatments.

By definition, ligand-dependent Wnt alterations can only induce downstream Wnt pathway activation in the presence of Wnt ligand. As a result, depletion and inactivation of Wnt ligand by inhibition of porcupine is a viable therapeutic approach for ligand-dependent tumors. In vitro models of ligand-dependent tumors, including organoids with RNF43 mutations [79] and cell lines with RSPO fusions [46], are exquisitely sensitive to porcupine inhibitors. This has also been demonstrated in various in vivo settings, including xenografts with RSPO fusions [77] and autochthonous Rnf43/Znrf3-null intestinal tumors [80]. Porcupine inhibition is associated with marked repression of Wnt pathway activity, reduced tumor size and substantial remodeling the transcriptomic landscape that includes increased intestinal differentiation [77,80]. Porcupine inhibitors have entered early-phase clinical trials (NCT01351103, NCT03447470, NCT03507998). Preliminary evidence from a phase 1 trial identified a partial response in one patient with a detectable RNF43 mutation [81] while porcupine inhibition was associated with reduced AXIN2 expression, suggesting on-target effects [82].

However, in vitro modeling of porcupine inhibition in ligand-dependent CRC cell lines has identified selection for resistance mutations, such as loss-of-function alterations to AXIN1, leading to loss of function of the destruction complex and downstream constitutive pathway activation [46]. It is worth noting that AXIN2 repression seen in ligand-dependent tumors does not result in downstream pathway activation because of redundancy with AXIN1. AXIN1 is a constitutive component of the destruction complex and not a Wnt pathway target. This would suggest that AXIN1 inactivation alone would not be sufficient to drive Wnt pathway activation unless AXIN2 was concurrently repressed—we would hypothesize that this situation could only arise in ligand-dependent tumors. This might explain the relatively low frequency (<0.05%) of truncating AXIN1 mutations seen in CRC [8].

In tumors with epithelial RSPO fusions, the autocrine signaling loop can be blocked by an anti-RSPO3 antibody. For example, treatment with anti-RSPO3 antibody has been shown to result in inhibition of xenograft tumor growth with tumor regression in some cases [27,83,84,85]. As with porcupine inhibitors, this was associated with evidence of increased intestinal differentiation on morphological and transcriptomic analysis [27,83]. This differentiated phenotype was associated with reduced expression of stem cell markers and key Wnt targets such as LGR5 and ASCL2. A phase 1 trial of an anti-RSPO3 antibody in patients with metastatic colorectal cancer was associated with partial responses in some patients, although this was not clearly associated with baseline RSPO3 expression [86]. In addition, while it has not been formally tested, it is entirely plausible that anti-RSPO3 therapy would also be effective for tumors with stromal RSPO overexpression.

The Wnt pathway plays a critical role in bone homeostasis [87] and unsurprisingly inhibition of ligand-dependent Wnt signaling via porcupine inhibitors or anti-RSPO3 antibodies results in on-target bone toxicity, including reduced bone strength and pathological fractures [86,88,89]. Consistent with this, porcupine-null mice have widespread bone defects, while germline loss-of-function Wnt ligand mutations in humans are associated with high fracture risk [90,91,92]. Preliminary evidence has shown that bone toxicity could be reduced with co-administration of denosumab, which inhibits bone degradation [89]. Altogether, concerns about resistance and on-target toxicity would likely limit the use of direct Wnt inhibitions (porcupine, anti-RSPO3) to short durations of time, likely in conjunction with other treatments.

In light of evidence that ligand-dependent tumors may depend upon repression of negative regulators, possibly via promoter hypermethylation [42], demethylating agents could be a viable therapeutic strategy in ligand-dependent tumors. Demethylation treatment with azacitidine in HCT116, a colorectal cancer cell line with an RNF43 mutation and comparatively low AXIN2 expression, resulted in increased AXIN2 expression and increased cell death [60,93]. Azacitidine is an approved treatment for myelodysplastic syndrome with a well-established toxicity profile suggesting that this would be a feasible treatment for ligand-dependent CRC [94].

Unexpectedly, recent work in acute myeloid leukemia found that asparaginase treatment was synthetically lethal with inhibition of GSK3 [95]. Asparaginase functions to deaminate and so degrade the nonessential amino acid asparagine, which is required for leukemic cell growth [96]. GSK3 mediates ubiquitination of a wide range of proteins, such as APC, and resulting proteasomal degradation provides a source of asparagine in the cell. Asparaginase treatment has a relatively favorable toxicity profile and is licensed for acute myeloid leukemia [97]. In contrast to ligand-independent alterations, which act downstream and by-pass GSK3, ligand-dependent mutations directly lead to inhibition of GSK3 (Figure 1) through activation of the canonical Wnt pathway, thus explaining a unique selective vulnerability for asparaginase treatment in ligand-dependent tumors. Specifically, asparaginase treatments were highly toxic for organoids with RSPO fusions but had no activity against organoids with APC or CTNNB1 mutations [98]. Treatment of mice with subcutaneous implantation of RSPO-mutant organoids was associated with marked tumor regression and prolonged progression-free survival, with no evidence of early therapy resistance [98]. No benefit was seen for implanted APC-mutant organoids. Altogether, these data would suggest that asparaginase could be a viable and well-tolerated treatment for patients with ligand-dependent CRC.

In summary, ligand-dependent Wnt biology is associated with a range of therapeutic vulnerabilities that could be exploited as effective anti-cancer therapy.

## 9. Combination Therapy for Ligand-Dependent Tumors

Considering that direct inhibition of the Wnt pathway is unlikely to be feasible for extended periods of time, it is important to consider how treatments for ligand-dependent tumors might synergize with existing anti-cancer therapy. Wnt pathway activation is often detected as a marker of resistance to cytotoxic chemotherapy [99]. Resistance to paclitaxel, which is a type of cytotoxic chemotherapy that inhibits microtubule detachment from centrosomes, is associated with Wnt pathway activation, detected as increased CTNNB1 protein expression. Considering that Wnt functions as a regulator of centrosome separation [100], it is feasible that Wnt activation could directly promote survival of tumor cells. Consistent with this, anti-Wnt treatments such as anti-RSPO3 antibodies synergize with paclitaxel in patient-derived xenografts with RSPO3 fusions [84].

More generally, inhibition of Wnt signaling in ligand-dependent tumors is consistently shown to skew cells from a stem-like phenotype to a more differentiated phenotype [27,101,102]. Resistance to cancer radiotherapy and chemotherapy is often driven by acquisition of stem-like phenotypes, with enrichment of tumor cells that are able to repopulate a tumor on transplantation, often termed cancer stem cells [103,104]. This suggests that short courses of Wnt pathway inhibitors could be synergistic with a wide range of existing and innovative drug regimens, especially if Wnt inhibitors are early in the treatment schedule.

The Wnt signaling pathway appears to play a role in protecting cells from immune surveillance. As a result, there is considerable interest in the combination of immunotherapy that targets PD-L1/PD-1 signaling and direct inhibition of the Wnt signaling pathway. Signaling through the PD-1 receptor is thought to promote an exhausted phenotype in cytotoxic T cells that impairs effective anti-tumoral immunity [105]. While immunotherapy has demonstrated activity in diverse tumor types, it has proved ineffective in unselected patients with CRC [5]. There are multiple lines of evidence that the Wnt signaling pathway can directly promote an immune suppressive environment [106]. Early data from trials of porcupine inhibitors have shown evidence for increased expression of activated immune signatures [82]. Furthermore, porcupine inhibition was synergistic with anti-CTLA4 immunotherapy in a murine melanoma model [107]. Altogether, this raises the question of whether anti-Wnt therapies would act synergistically with immunotherapy in colorectal tumors and this hypothesis is under active investigation in several early-phase clinical trials (NCT01351103, NCT02521844, NCT02675946).

Furthermore, as discussed above, the microsatellite-unstable subset of colorectal tumors ligand-dependent tumors is enriched with tumors, which have enhanced responses to immunotherapy [6]. Unexpectedly, a recent analysis incorporating a large cohort of patients with colorectal cancer who were treated with immunotherapy, demonstrated that RNF43-mutant tumors responded significantly better to immunotherapy than would have been expected from their mutational burden [108]. This is an exciting finding that raises the possibility the ligand-dependent Wnt biology might be independently associated with responses to immunotherapy and warrants further investigation in additional cohorts.

## 10. Outlook—Landscape of Precision Medicine in CRC

Approximately 15% of colorectal tumors have ligand-dependent alterations in the Wnt signaling pathway, affecting RNF43 or RSPO2/3. This unique Wnt biology is associated with a range of specific therapeutic vulnerabilities, especially to depletion of Wnt ligand by porcupine inhibitors. There is a strong theoretical basis for the combination of immunotherapy with a time-limited course of porcupine inhibition. Inhibitors of ligand-dependent Wnt signaling are known to be ineffectual in tumors with ligand-independent alterations such as APC mutations [79]. As a result, due to the low frequency of ligand-dependent alterations, clinical trials of these selective treatments will fail in unselected patients. Precision medicine depends upon the ability to stratify patients into clinically meaningful subsets, followed by targeting with biologically appropriate therapies. It is contingent on the existence of biomarkers specific for each subset that can be feasibly adopted into routine clinic practice. We propose that AXIN2 is one such biomarker and could be measured at low cost from routine clinical specimens. It can be measured by high-throughput qRT-PCR and does not require costly and time-consuming DNA and RNA sequencing. On the basis of AXIN2 expression, it would be possible to identify patients with ligand-dependent Wnt biology who could then be targeted with effective personalized therapies. In summary, we propose that the concept of ligand-dependent tumors as an individual disease entity has the potential to revolutionize precision medicine and improve the outcomes for patients with colorectal cancer.

## Figures and Tables

**Table 1:** Driver Wnt alterations in colorectal cancer. Prevalence refers to the frequency of each mutation in the subset of colorectal tumors with a detectable driver Wnt alteration, as derived from [42]. Loss-of-function alterations in APC and RNF43 are frequently accompanied by loss of heterozygosity (LOH) affecting the second allele [13].

Mutation Type	Gene	Type of Alteration	Prevalence in CRC
Ligand-dependent	RNF43	Loss-of-function	10%
		- Nonsense	
		- Frameshift	
	R-spondin (RSPO2, RSPO3)	Gain-of-function	8%
		- Stromal overexpression	
		- Epithelial gene fusions	
Ligand-independent	APC	Loss-of-function	81%
		- Nonsense	
		- Frameshift	
	CTNNB1	Gain-of-function	2%
		- Missense (affecting codons 31–35, 37, 40, 41, 45, 383 and 387)	
